# A Review on the Mechanism, Impacts and Control Methods of Membrane Fouling in MBR System

**DOI:** 10.3390/membranes10020024

**Published:** 2020-02-04

**Authors:** Xianjun Du, Yaoke Shi, Veeriah Jegatheesan, Izaz Ul Haq

**Affiliations:** 1 College of Electrical and Information Engineering, Lanzhou University of Technology, Lanzhou 730050, China; yaoke_shi@163.com (Y.S.); Izaz.lut@gmail.com (I.U.H.); 2 School of Engineering, RMIT University, Melbourne 3000, Australia; jega.jegatheesan@rmit.edu.au; 3 Key Laboratory of Gansu Advanced Control for Industrial Processes, Lanzhou University of Technology, Lanzhou 730050, China; 4 National Demonstration Center for Experimental Electrical and Control Engineering Education, Lanzhou University of Technology, Lanzhou 730050, China

**Keywords:** membrane fouling, influencing factors, control method

## Abstract

Compared with the traditional activated sludge process, a membrane bioreactor (MBR) has many advantages, such as good effluent quality, small floor space, low residual sludge yield and easy automatic control. It has a promising prospect in wastewater treatment and reuse. However, membrane fouling is the biggest obstacle to the wide application of MBR. This paper aims at summarizing the new research progress of membrane fouling mechanism, control, prediction and detection in the MBR systems. Classification, mechanism, influencing factors and control of membrane fouling, membrane life prediction and online monitoring of membrane fouling are discussed. The research trends of relevant research areas in MBR membrane fouling are prospected.

## 1. Introduction

Membrane bioreactors (MBRs) combine both biological treatment and physical separation (using membranes) of various pollutants to treat domestic and industrial liquid wastes. Due to the combination of the above-mentioned processes, MBRs produce treated effluents of higher quality compared to the conventional activated sludge process [1]. MBRs are simple to operate when experienced operators are employed and produce less sludge. They also have a very small footprint which is very valuable when MBRs are installed in dense urban areas where space is at a premium. The advantages mentioned above, along with the ever-decreasing cost of membrane materials and the increasingly stringent requirements of treated effluent quality, mean that MBR technology is more and more widely applied in wastewater treatment [2]. However, membrane fouling affects the operating flux and the life of membranes. There is no unified statement about the mechanisms of membrane fouling, but from the analysis of the causes of membrane fouling, the following mechanisms of membrane fouling in MBR have been proposed and verified: (i) narrowing of membrane pores; (ii) the adsorption of the solute in the solution by the membrane [3]; (iii) the deposition of the (activated) sludge floc on the membrane surface [4]; and (iv) the compaction of the filter cake layer on the membrane surface. These mechanisms alone or together play a leading role at different stages of the membrane filtration process. As various factors govern the operating cost of a membrane system, such as power requirements, costs of power, labor, materials, membrane cleaning, scale inhibition and membrane life and replacement, some limitations remain in using membranes for water and wastewater treatment [5]. Once membrane fouling occurs, it will reduce permeate flux, increase feed pressure, reduce productivity, increase system downtime, increase membrane maintenance and operation costs due to membrane cleaning, and decrease the lifespan of the membrane modules [6]. Thus, the main challenge in the application of MBRs is to find a solution to the fouling of membranes [7,8]. Most of the existing papers on membrane fouling review a specific kind of research direction in a specific area (see Appendix A), which is not comprehensive enough. However, membrane fouling itself involves a wide range of content and covers a lot of knowledge basics. Those who want to promote the research on membrane fouling must fully understand membrane fouling. In this paper, the traditional research content of membrane fouling and the latest research results are integrated together, including every aspect of membrane fouling research by many scholars and researchers in the past decades, which implements the most complete discussion of influencing factors, mechanisms and control methods of membrane fouling by far. It provides a more comprehensive and systematic reference for follow-up studies on membrane fouling and relevant areas.

## 2. Classification of Membrane Fouling

Membrane fouling can be classified into internal fouling, external fouling and concentration polarization fouling. The fouling caused by the deposition as well the adsorption of solutes and colloidal particles on the interior of the membrane pores is called internal fouling and sometimes referred to as pore blocking [9]. The deposition of particles, colloids and macromolecules on the membrane surface is called external fouling. External fouling forms a fouling layer on the membrane surface. The fouling layer can be classified as gel layer or cake layer. The gel layer is formed by the deposition of macromolecules, colloids and inorganic solutes on the surface of the membrane due to the pressure difference between the feed and permeate sides of the membrane. The cake layer is formed by the accumulation of solids on the membrane surface [10]. Concentration polarization refers to the accumulation of solutes and ions in the thin liquid layer adjacent to the membrane surface [11], which is an inherent phenomenon in the membrane filtration process. Concentration polarization increases the flow resistance and decreases the membrane flux. The concentration polarization layer is determined by the convective shear force [12]. Increasing the convective velocity can alleviate membrane resistance caused by concentration polarization.

Membrane fouling has traditionally been divided into reversible fouling and irreversible fouling according to the degree of removal of foulants [13]. Reversible fouling refers to the part of the foulants that can be removed by physical means such as backwashing or intermittent operation of membranes under cross-flow filtration. Non-reversible fouling refers to the fouling that needs chemical cleaning and cannot be removed by physical cleaning [14]. It is generally believed that reversible fouling is caused by loose deposition of contaminants on the surface of the membrane and irreversible fouling is caused by the blockage of membrane pores and strong adhesion of contaminants to the surface of the membrane. Many studies have pointed out that the formation and compaction of the cake layer is the main form of membrane fouling compared to membrane pore blocking [15]. Table 1 shows the onset of various fouling of membranes in an MBR.

According to the composition of pollutants, membrane fouling can be divided into organic fouling, inorganic fouling and biofouling. Organic fouling is caused by organic macromolecules. Wang et al. [16] found that organic macromolecular polymer clusters (BPCs) are important contaminants. Analysis shows that BPCs are less than 50 µm in diameter, which are significantly different from activated sludge floc particles. Lin et al. [17] found that the organic matter content in the activated sludge supernatant was significantly higher than that in the MBR effluent, and the high content of organic matter in the supernatant was considered to contain BPCs. BPCs act like glue, which helps the sludge to adhere to the surface of the membrane and form a cake layer. Wang et al. [18] studied the formation process and fouling characteristics of dynamic membranes as well as an improved self-forming dynamic membrane bioreactor (SF-DMBR) for the recovery of organic matter in wastewater and to evaluate its properties. The results showed that 80% of the organic matter in the wastewater can be recovered faster in the case of continuous operation. Inorganic foulants include struvite, K_2_NH_4_PO_4_ and CaCO_3_. Biofouling is caused by the interaction between biological substances and membranes. Gao et al. [19] found that about 65% of the particles in the membrane cake layer are smaller than the pore size of the membrane (0.1–0.4 µm), so that they can pass through the membrane pores and can block the membrane pores. Biofouling also includes adsorption of extracellular polymers (EPS) and microbial metabolites (SMP), which are produced by microbial secretion, onto the membrane surface and membrane pores [20]. The microbial colony structures in the membrane cake layer and in the mixed liquor are significantly different. Some strains will preferentially adsorb onto the membrane surface due to the secretion of more EPS, resulting in serious biofouling [21].

## 3. Factors Affecting the Fouling of Membranes

There are many factors that cause membrane fouling, including the material of the membrane module, the pressure difference across the membrane during filtration, the cross-flow velocity, the hydraulic retention time (HRT), the sludge retention time (SRT), microbial polymerization and dissolution processes, and mixing [22]. These factors alone or in combination provide conditions for membrane fouling, or contribute directly or indirectly to membrane fouling. Understanding and mastering the effects of various factors on membrane fouling is essential to prevent, control and predict membrane fouling [23]. The influencing factors on membrane fouling are shown in Table 2.

### 3.1. Influence of Membrane Intrinsic Properties on Membrane Fouling

Intrinsic properties of the membrane that affect membrane fouling include material, hydrophilicity/hydrophobicity, surface charge, roughness, pore size, porosity, and structure of the membrane module.

#### 3.1.1. Effect of Membrane Material on Membrane Fouling

Commonly used membranes are mainly classified into organic membranes, ceramic membranes, and metal membranes according to the type of materials used to synthesize membranes. Among them, the organic membranes have a low cost and mature manufacturing process, and are currently the most widely used [24]. However, they have low strength as well as a short life and are easily fouled. Common organic membranes include polyethylene (PE), polysulfone (PS), polyethersulfone (PES), polyacrylonitrile (PAN) and polyvinylidene fluoride (PVDF). Among them, PVDF membrane has a higher anti-fouling ability. Compared with organic membranes, ceramic membranes and metal membranes have (i) better mechanical properties, (ii) resistance to high temperatures, and (iii) high flux; however, these two materials are difficult to manufacture and are expensive [25]. In the case of the same operating conditions [26], the PAN membrane pollutes slower than the PES membrane. Researchers [27] found that metal membranes are easier to recover from fouling than organic membranes.

#### 3.1.2. Effect of Hydrophilicity/Hydrophobicity on Membrane Fouling

With the continuous application of membrane bioreactors, membrane fouling has become a major bottleneck limiting its further development [28,29]. At the beginning of operation, hydrophilic organic matter will be the dominant pollutant; however, the interaction force between hydrophobic organic matter and the membrane is significantly greater than the interaction force between hydrophilic organic matter and the membrane, resulting in hydrophobic organic matter becoming a dominant pollutant in the later stage of operation [30]. Among them, hydrophilic carbohydrate organic matter and hydrophobic humic organic matter abundantly present in the wastewater to be treated are key substances causing membrane fouling [31]. Therefore, it is important to find out how the pro-/hydrophobic organic matter contaminates the membrane to prevent and control the membrane fouling.

The hydrophilicity/hydrophobicity of the membrane is usually characterized by the contact angle θ. The larger the value of θ, the stronger the hydrophobicity of the membrane surface. The value of θ angle has a certain relationship with the morphology of the membrane surface and the pore size of the membrane. The hydrophilicity/hydrophobicity of the membrane material has a great influence on the anti-fouling performance of the membrane [32]. The hydrophilic membrane is less affected by adsorption, has a larger membrane flux, and has superior anti-fouling properties compared to the hydrophobic membrane. However, some researchers have concluded that the most hydrophilic PES membrane suffers from the most serious membrane fouling, which may be related to the maximum membrane pore opening of PES. It is worth noting that the hydrophilicity/ hydrophobicity of the membrane usually only has a significant effect on membrane fouling at the initial stage of filtration. After the initial fouling, the chemical properties of the foulants will replace the chemical properties of the membrane itself as the main influencing factor [33].

The natural organic matter present in wastewater is divided into strongly hydrophobic, weakly hydrophobic, polar hydrophilic and neutral hydrophilic organic matter, and is filtered separately. It is found that the most important organic matter causing the decrease of membrane flux is neutral hydrophilic organic matter. It is believed that the presence of more hydrophilic organic matter in the raw water causes more serious membrane fouling [19]. However, some researchers have reached the opposite conclusion [15,34]. Their experiments showed that the hydrophobic organic matter is the main factor causing the decline in flux.

#### 3.1.3. Effect of Membrane Surface Charge on Membrane Fouling

When the membrane surface charge is the same as the charge of the pollutants present in the wastewater, it can improve the membrane surface contamination and increase the membrane flux. In general, the colloidal particles in the aqueous solution are negatively charged. So, if the material having a negative potential is used as the membrane material, it can prevent membrane fouling due to the repellent effect of similar charges.

**Figure 1:**
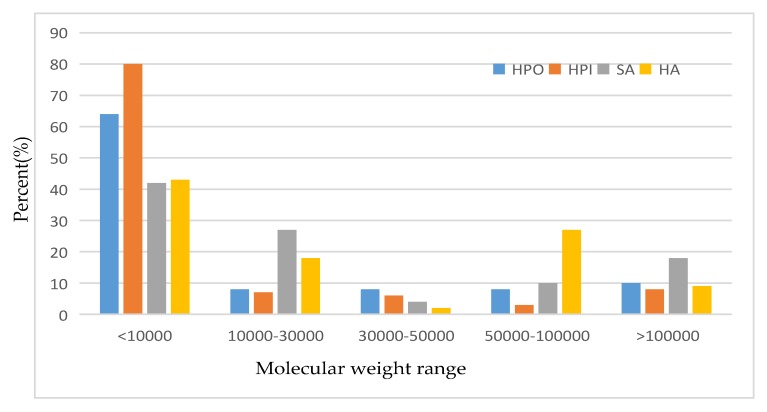
Molecular weight distribution of different organic matters in secondary treated effluent. (Note: HPO-hydrophobic; HPI-hydrophilic; SA-sodium alginate; HA-humic acid).

Lin et al. [35] investigated the effects of operating pressure difference, cross-flow rate, feed solution concentration and operating temperature on the flow potential of suspended-growth (SG) nanofiltration membranes; the membranes were filtering NaCl, Na_2_CO_3_ and CuCl_2_ solutions and they found that SG membranes have negative surface charges. At the same time, the absolute values of electric density, zeta potential and charge density increased with the increase of cation or anion valence state. The higher the operating pressure difference and the concentration of the liquid solution, the lower the velocity of the cross flow and the influence of the valence state of the ions. The cation of the same valence state have a greater effect on the charge performance of the membrane than the anions [36]. Figure 1 shows the molecular weight distribution range of the simulated hydrophilic (HPI) and hydrophobic (HPO) organics in the secondary treated effluent.

As can be seen from the figure, the actual molecular weight distribution range of hydrophilic/hydrophobic organics is relatively small, mainly concentrated in the range of <10,000 Dalton. The proportion of organic molecules with HPO < 10,000 Dalton is close to 70%, and especially the proportion of organic molecules with HPI < 10,000 is up to 80%.

#### 3.1.4. Effect of Membrane Pore Size, Distribution and Structure on Membrane Fouling

**Figure 2:**
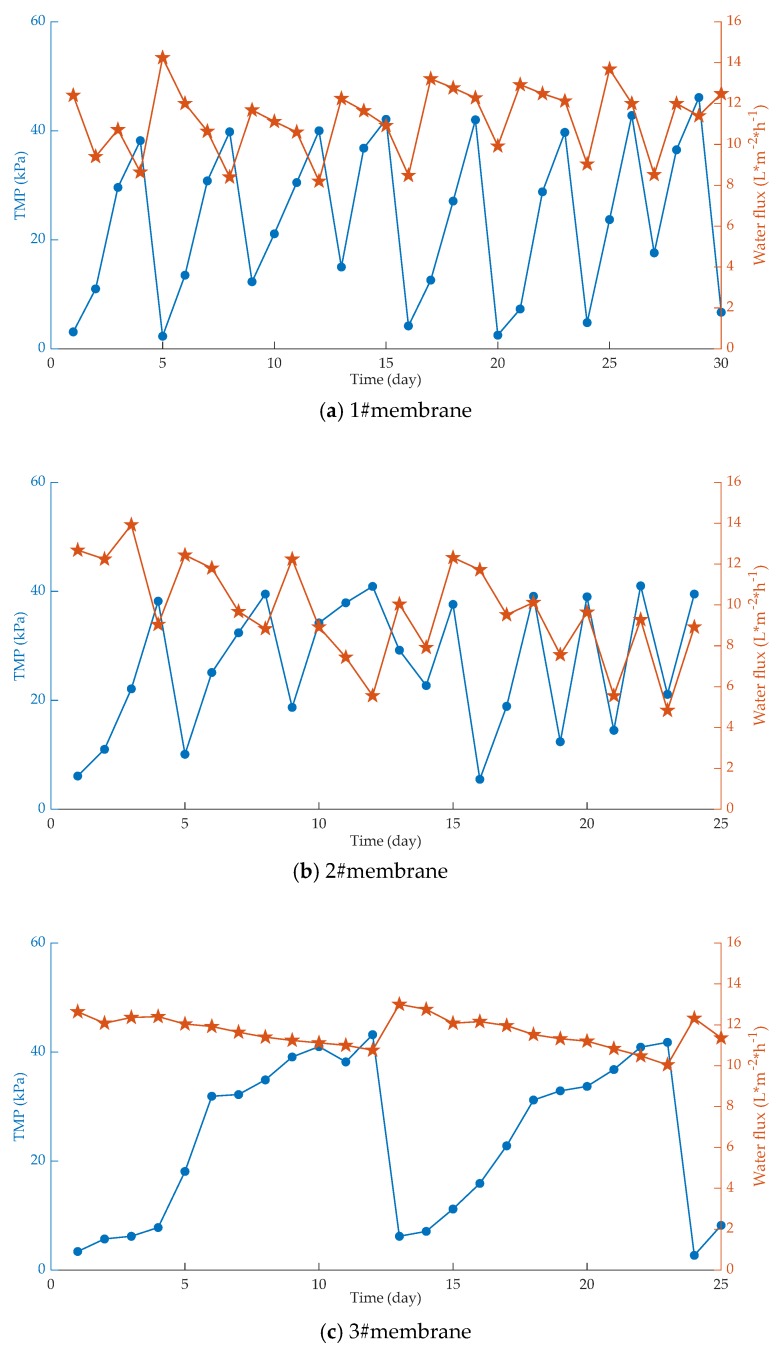
Temporal variations of flux and transmembrane pressure (TMP) when membranes with different pore sizes are used. (**a**) 1#membrane; (**b**) 2#membrane; (**c**) 3#membrane.

In a treatment study of wastewater containing micropollutants [37], it was found that the pore size of the membrane is the main factor affecting the membrane flux and the turbidity removal rate of the wastewater. The larger the membrane pore size, the more serious the fouling of the membrane, and faster the flux decay as well as the lower the removal rate. Membrane pore size is an important indicator that directly affects the separation performance of the membrane. In the membrane filtration process, membrane surface or membrane pores easily become sites for adsorption and deposition; they are also blocked by tiny particles or solute macromolecules present in water [38]. According to the characteristics of water sources with micropollutants, the membrane fouling caused by small molecular substances cannot be ignored. When using MBR to treat water with micropollutants, it is important to select membranes with appropriate pore size for process operation and membrane fouling control. The change of transmembrane pressure difference and water flux with time in three kinds of membranes with different pore sizes is shown in Figure 2. As the pore size of the membrane increases, the transmembrane pressure difference of the membrane increases rapidly [39].

It is found from Figure 1 that the degree of membrane fouling is aggravated with the extension of the running time, resulting in an increase in the transmembrane pressure difference and a decrease in the water flux. In addition, the smaller the membrane pore size, the slower the increase in transmembrane pressure difference, the longer the membrane cleaning cycle time, the larger the membrane pore size, the more serious the membrane fouling, and the shorter the membrane cleaning cycle time. However, due to high membrane surface porosity and fiber-interwoven network-like pore structure, (i) the membrane can be cleaned effectively, (ii) the performance of the membrane can be recovered very well after repeated cleaning, and (iii) the water flux of the membrane changes minimally [40].

#### 3.1.5. Effect of Porosity and Roughness on Membrane Fouling

Membrane porosity and roughness also have a potential impact on membrane fouling behavior. Generally, the larger the porosity, the smaller the transmembrane pressure (TMP). However, as the porosity changes, the surface properties of the membrane, such as roughness, also change. This in turn changes the possibility of adsorbing contaminants on the membrane surface. The organic membrane porosity is usually higher than that of the inorganic membrane, but the flux is often lower than that of the inorganic membrane [41,42]. When the membrane surface roughness is large, the membrane is more susceptible to fouling [43].

#### 3.1.6. Effect of Membrane Module Structure on Membrane Fouling

**Figure 3:**
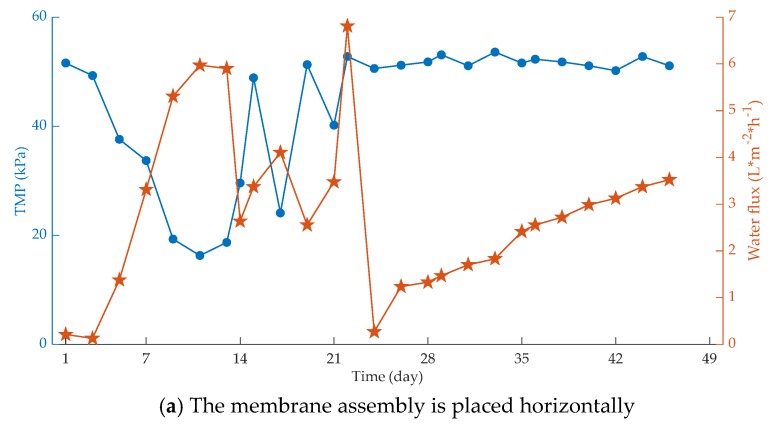

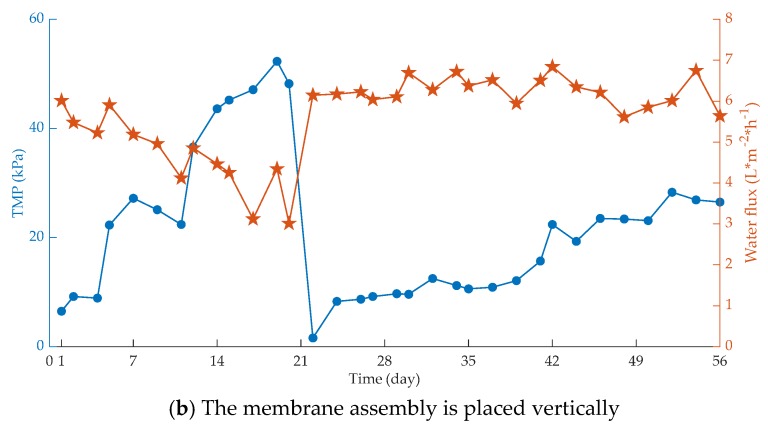
Influence of membrane placement on membrane fouling. (**a**) The membrane assembly is placed horizontally; (**b**) The membrane assembly is placed vertically.

The membrane module is the core of membrane separation technology. For a membrane separation process, not only membranes with excellent separation characteristics but also membrane modules and devices with compact structure and stable performance must be applied in large-scale industrial processes [44]. In the process of studying PVC membrane materials [45], it was found that under the same operating conditions, the membrane bioreactor with a vertical membrane module has a better flow state, and the shearing effect of the gas and liquid two-phase flow generated by aeration and scouring is much stronger than that of a horizontal device. In addition, the bottom of the horizontal device is perforated and aerated, the PVC film is thicker, and the sludge deposition on the side of the aeration hole is also serious, so the membrane fouling rate is faster and the corresponding operation period is shorter [46]. Under the same operating conditions, the impact of different placement modes on membrane fouling of two typical systems is shown in Figure 3.

It can be seen that under the same operating conditions, the flow condition of the membrane bioreactor with the membrane component placed vertically is better, and the shear effect of gas–liquid two-phase flow generated by aeration scour is much stronger than that of the horizontal release device.

**Figure 4:**
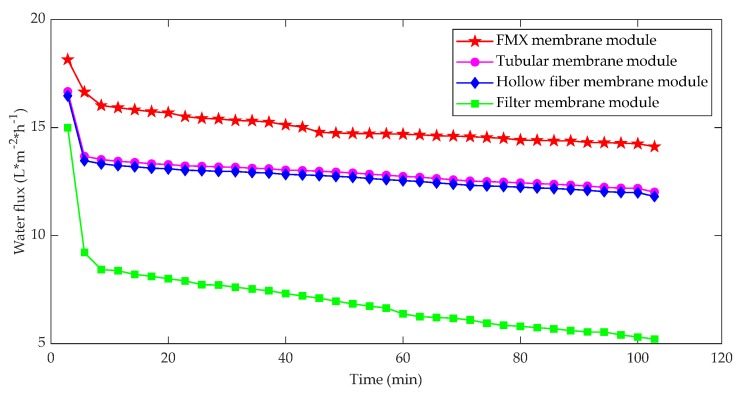
Variation of permeate flux of four membrane modules.

Since FMX maintains high flux and recovery rate under the most challenging conditions, it has been used for wastewater treatment, separation and dewatering in manufacturing processes and recovery applications in various industries. Chen et al. [47] compared the membrane filtration process using FMX rotating disc plate, hollow fiber, tubular and filter cup membrane modules; the results showed that the FMX membrane module yielded higher membrane flux with lower fouling of the membrane, followed by the hollow fiber membrane, tubular, and the filter cup membrane modules when operated under dead-end filtration mode. Results are shown in Figure 4.

### 3.2. Effect of Operating Conditions on Membrane Fouling

Operating conditions affecting membrane fouling include membrane flux, TMP, aeration, cross-flow velocity (CFV), SRT, HRT, and temperature. Furthermore, mode of operation, including influent water quality and sludge loading, directly affects membrane fouling factors.

#### 3.2.1. Effect of Membrane Flux and TMP on Membrane Fouling

In membrane filtration operations, membrane flux and TMP are two quantities associated with each other [48]. If other conditions remain the same, in order to obtain higher membrane flux, TMP must be increased. Conversely, if TMP is increased or decreased, membrane flux will change accordingly [49]. MBR has two modes of operation: constant flux and constant pressure. Many studies have demonstrated the existence of critical fluxes; MBRs are operated below critical to avoid excessive fouling of the membrane during the initial phase of operation [50]. Under low-pressure operation, the initial filter cake layer formed is thin or only has a reversible concentration polarization layer, so the membrane fouling is not significant. However, operating the MBR above the critical TMP makes the initial filter cake layer thicker or forms a concentrated polarization layer. It is also converted into a dense filter cake layer [51], which increases membrane fouling. Lowering the initial TMP can reduce membrane fouling and slow down the rate of membrane flux decline [52]. In addition, membrane fouling is relatively slow in constant flux operation compared to constant TMP operation, but membrane flux recovery is poor after cleaning. This may be due to the continuous densification of the fouling layer on the membrane surface during constant flux operation [53].

#### 3.2.2. Effect of Aeration and CFV on Membrane Fouling

Aeration is an important parameter in the operation of MBR. It not only provides the oxygen necessary for metabolism of activated sludge, but also washes the surface of the membrane, avoids the deposition of pollutants and slows the fouling of the membrane. Therefore, membrane bioreactors often use relatively larger aeration [54]. The change of aeration volume in MBR will cause changes in the characteristics of effluent water quality and sludge mixture. When the aeration rate increases, the effluent quality will improve and the removal rates of COD (chemical oxygen demand) and NH_4_^+^-N will increase, but the sludge floc size will decrease, the impact on sludge concentration and sludge load is weak. As the amount of aeration increases, the total amount and composition of SMP will change [55]. Among them, the protein/polysaccharide value has an important influence on the sludge properties, which in turn affect the physical, chemical and biological properties of the sludge mixture and ultimately affect the fouling rate. The occurrence of membrane fouling will lead to a decrease in membrane flux to some extent [56]. Moderate aeration can reduce membrane fouling and increase membrane flux to some extent [57]. Zhang et al. [58] found that when the aeration intensity increased to a certain extent, membrane pore adsorption, clogging and membrane gel layer resistance became the main membrane resistance and the fouling rate increased. Therefore, there is an optimum aeration intensity for the operation of MBR.

The change in CFV changes the diffusion caused by shear to affect the migration of particles from the surface of the membrane, which in turn affects the thickness of the cake layer. The membrane flux increases approximately linearly with increasing CFV. However, the CFV is not as a large factor as the aeration, and the better the membrane filtration performance will be after CFV exceeds the critical value. However, when the CFV exceeds a certain threshold, the TMP will increase as the CFV increases [59]. This is because higher CFV reduces the deposits of larger particles and allows the filter cake layer to consist primarily of small particles. Those particles are more compact and lead to higher TMP. Moreover, too large a CFV will cause the sludge particles to break, which will make the filter cake layer more dense, and also stimulate the release of EPS and increase membrane fouling [60].

#### 3.2.3. Effect of SRT and HRT on Membrane Fouling

SRT can affect MLSS (mixed liquid suspended solids), sludge composition, EPS and other parameters which are important operating conditions affecting membrane fouling rate in an MBR. HRT has an indirect effect on membrane fouling. First, changes in HRT will directly lead to changes in membrane flux, which in turn will change the state of membrane filtration and affect the rate of membrane fouling [61].

**Figure 5:**
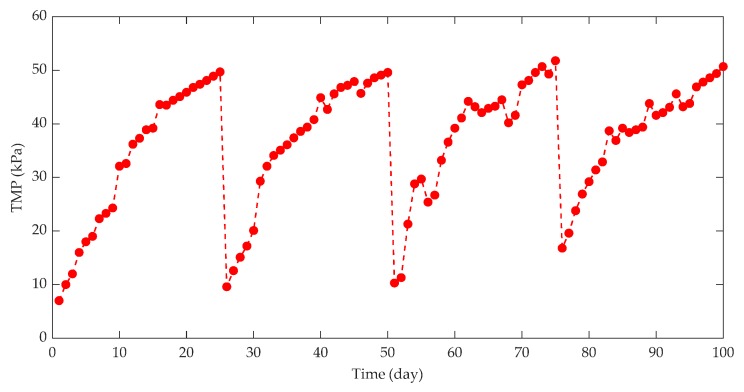
TMP at different hydraulic retention times (HRT).

When investigating the effect of HRT on membrane fouling in a split-type anaerobic membrane bioreactor (AnMBR) treating beer wastewater, it was found that shorter HRT can produce higher OLR and F/M, which in turn affects the metabolic activities of anaerobic microorganisms and microbial metabolites (the content of EPS and SMP), and sludge particle size increases, resulting in serious membrane fouling [62]. The change of TMP is an important indicator that directly reflects the membrane fouling of an AnMBR, and the influence of HRT on TMP is shown in Figure 5.

This indicates that shorter HRT will lead to rapid increase of TMP and aggravate membrane fouling, which is not conducive to long-term stable operation of AnMBR.

Sludge properties have an important effect on membrane fouling [63]. The rate of membrane flux decline is positively correlated with the ratio of protein to polysaccharide, sludge settling performance and relative hydrophobicity, but negatively correlated with EPS [64]. With the extension of SRT, the total amount of EPS in the mixture showed a decreasing trend. The ratio of protein to polysaccharide in tightly bound extracellular polymeric substances increased, the sedimentation performance of sludge became worse, the relative hydrophobicity increased, the rate of membrane flux decreased, and membrane fouling aggravated [65]. At the same time, a large number of studies have shown that with the appropriate extension of SRT, MLSS increases, SMP concentration decreases, and membrane fouling is somewhat relieved. Therefore, the MBR system has an optimal SRT value.

#### 3.2.4. Effect of Temperature on Membrane Fouling

Temperature changes affect the enzyme activity, mass transfer rate and microbial activity of anaerobic microorganisms. Changing the viscosity of the liquid can affect the treatment efficiency and stability of the reactor [66]. A study on the effect of temperature on the treatment effect and membrane fouling of the anaerobic membrane bioreactor [67] found that the microorganisms in the anaerobic membrane bioreactor can maintain high activity and COD removal rate at higher temperatures. In addition, the temperature will significantly affect the metabolism of microorganisms, resulting in different amounts of EPS secretion. At low temperatures, microorganisms secrete more polysaccharides and proteins for self-protection, resulting in higher EPS [68]. This information can help to optimize the anaerobic membrane bioreactor. The operating temperature should be found such that the system EPS concentration is at a lower level and therefore slowing down the fouling of the membrane [69].

Generally, the increase in temperature will decrease the viscosity of the mixed liquor in the MBR, (i) increase the solubility of suspended particles, (ii) increase the mass transfer diffusion coefficient [70], (iii) promote the movement of solute on the membrane surface to the bulk solution, (iv) reduce the thickness of concentration polarization layer, so as to improve the cross-flow velocity, and (v) increase the flux of the membrane [71].

When studying a forward osmosis membrane bioreactor, temperature has a significant effect on the forward osmosis process. The water flux increases with increasing temperature. The influence of the temperature of the draw solution is significantly higher than that of the feed solution, and only the temperature of the draw solution is increased, which can obtain higher water flux and lower membrane fouling under the premise of significantly reducing the heat consumption of the system, which is an efficient operation mode [72].

#### 3.2.5. Effect of the Mode of Operation on Membrane Fouling

Among the many factors affecting membrane flux, the mode of operation is the key to the successful application of membrane technology in wastewater treatment. The cross-flow velocity can control the size of the permeate flux and to some extent contain membrane fouling [73]. The dynamic fouling layer formed by the pollutants on the surface of the membrane is the main reason for the decrease in membrane flux and the cross-flow filtration can effectively reduce the thickness of the dynamic fouling layer [74]. In cross-flow filtration, the addition of a gas such as nitrogen at the inlet end of the membrane module can increase the degree of turbulence in the pores and thereby enhance the shear-carrying effect on the fouling layer on the membrane surface, which will increase the flux. Increasing the cross-flow velocity helps to increase the permeate flux. However, as the cross-flow velocity increases, the increase in permeate flux decreases. Therefore, there is an optimal cross-flow velocity under a certain operating pressure, and the fouling is intensified above the optimal cross-flow velocity, resulting in flux attenuation [75].

For pressure-driven membrane filtration processes, operating pressure is the most direct factor. There is a critical operating pressure during operation. Exceeding the critical pressure causes the membrane to foul extremely seriously [76]. At higher operating pressures, the fine particles in the wastewater collect toward the inner surface of the membrane under high pressure, and the velocity is faster than the speed of the particles leaving the membrane surface, and particles form a contaminated layer on the membrane surface, causing rapid decay of flux. In addition, the increase in air volume is not proportional to the increase in flux [77]. As the amount of compressed air increases, the permeate flux does not increase and decrease. Therefore, it is also important to select the proper amount of air. On the one hand, it can reduce the fouling of the membrane surface. Increasing the permeate flux, on the other hand, does not cause unnecessary waste of energy consumption [78].

In addition, the gas/water two-phase flow can increase the degree of turbulence in the pores, thereby enhancing the shear-carrying effect on the fouling layer on the membrane surface, and effectively suppressing membrane fouling, thereby increasing the permeate flux [79]. Intermittent pumping is also an effective measure to delay the development of membrane fouling [14]. When the system is off, the TMP becomes zero and the back diffusion rate of foulants on the membrane surface is accelerated; the removal of back-diffused foulants near the membrane surface is also enhanced, and the membrane fouling is alleviated. However, the stoppage time cannot be too long. After stopping time reaches a certain level, the removal effect of membrane fluid scouring on membrane fouling will be greatly weakened, and the excessive pumping time will greatly reduce the system’s water production, so it should determine the optimal pumping time ratio based on actual conditions [80].

### 3.3. Effect of Character of Activated Sludge Mixture on Membrane Fouling

The activated sludge characteristics affecting membrane fouling include sludge components, MLSS, sludge viscosity, environmental conditions (pH, DO), EPS, SMP, inorganic matter and microbial communities present in the activated sludge [81]. Excessive growth of activated sludge, especially overgrowth of filamentous bacteria, can lead to serious membrane fouling, which makes the frequency of membrane chemical cleaning higher and increases operating costs.

#### 3.3.1. Effect of Activated Sludge Components on Membrane Fouling

**Figure 6:**
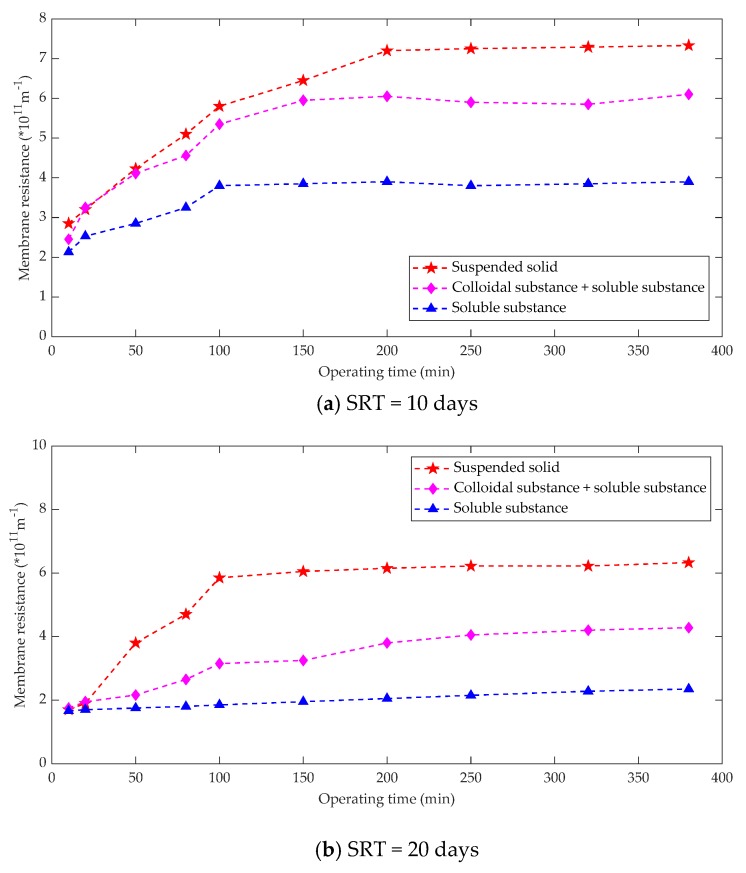

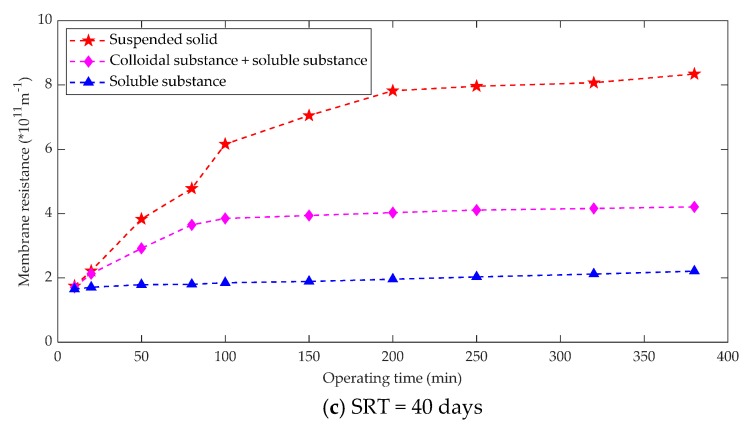
Relation between sludge composition and membrane fouling. (**a**) SRT = 10 days; (**b**) SRT = 20 days; (**c**) SRT = 40 days.

The sludge mixture consists of three components, namely suspended solids, colloids and dissolved matter. Membrane fouling is the result of a combination of all the above three components [82]. The sludge mixture of an anaerobic membrane bioreactor has small particles and the resistance formed by the suspended solids of the sludge mixture accounts for 70% of the total resistance, the resistance due to the colloidal substance accounts for 22%, and the resistance of the dissolved substance accounts for 8% [83]. For an anaerobic membrane bioreactor, it is advisable to adopt membrane fouling control measures such as increasing the air used for mixing to increase the flushing strength of the membrane surface and adding coagulants to increase the suspended solids from the viewpoint of reducing the suspended solids resistance in the sludge mixture [84]. Membrane fouling control measures could also optimize the size of particles in the activated sludge. The relationship between sludge components and membrane fouling under different operating conditions was measured as shown in Figure 6.

Some studies [85] summarized the contribution of different sludge components to membrane fouling in MBR operations. Obviously, there are major differences between the research results, which can be attributed mainly to the following aspects: matrix conditions, membrane filtration performance, hydraulic conditions, SRT, biological state and component separation methods. The sum of the three kinds of filtration resistance calculated after the component separation of the mixture is usually greater than the direct filtration resistance of the mixture, which is the result of the fact that direct filtration of the mixture is conducive to the formation of dynamic layer and the development of reducing membrane fouling.

In the membrane fouling process of an integrated membrane bioreactor, a mud cake layer will be formed on the surface of the membrane, the resistance will increase, and the membrane flux will decrease. The formed mud-cake-layer resistance is related to the particle size. The smaller the particle size, the smaller the porosity of the mud cake layer, and the greater the resistance; the larger the particle size, the larger the porosity of the mud cake layer and the smaller the resistance of the mud cake layer [86]. When wastewater is treated anaerobically, microorganisms rely mainly on secreted extracellular enzymes to decompose macromolecular proteins, fats and polysaccharides into small molecules, and then degrade small molecules to produce CH_4_ and CO_2_. The sludge formed is relatively loose, and the size of suspended particles in the mixed liquor is small [66].

#### 3.3.2. Effect of MLSS on Membrane Fouling

In the application of a membrane bioreactor, MLSS is an important process parameter which directly affects the performance of the membrane [46]. On the one hand, the high MLSS concentrations can reduce the sludge loading rate, improve the treatment efficiency and increase the viscosity of the mixed liquor; on the other hand, it can result in the increase in membrane filtration resistance. At the same time, the increase of sludge mass concentration will cause hypoxia or anaerobic phenomenon at the bottom of the reactor. In addition, the sludge concentration will increase and the aeration amount will remain unchanged, resulting in an anoxic state inside the activated sludge and short-range nitrification and denitrification in the reactor, so that the total nitrogen has a good removal rate [87].

The sludge mass concentration gradually increases with the increase of sludge age. At the beginning of the operation of an MBR, the sludge mass concentration increases greatly, mainly because of the strong microbial metabolism and high sludge load during this period. After that, the sludge growth is slow but the sludge mass concentration increases rapidly until the middle stages of operation. In the later stage, the sludge mass concentration is limited by organic pollutants, and after the sludge mass concentration reaches a certain level, it no longer grows and gradually reaches a stable state [88]. When the sludge mass concentration is stable, the COD of the supernatant and effluent will fluctuate with the COD in the raw wastewater, but the total removal rate of all the solutions’ COD and removal rate of the supernatant COD will no longer show significant fluctuations. It shows that the system is in stable state and has good removal effect on organic pollutants [89].

Under the condition that the flow rate of the water is kept constant, as the mass concentration of the sludge increases, the viscosity of the mixed liquid also increases, causing serious membrane blockage, resulting in a decrease in membrane porosity, thereby increasing filtration resistance and increasing TMP [90]. Studies have shown that the higher the sludge mass concentration, the greater the membrane filtration resistance. Although higher MLSS can increase the volumetric load of the membrane bioreactor, the increase of membrane resistance will increase energy consumption and therefore the operating costs, and affect effluent quality. Thus, the MLSS of the membrane bioreactor should not be too high, and should be considered in terms of treatment efficiency and processing capacity [33].

#### 3.3.3. Effect of Sludge Viscosity on Membrane Fouling

Viscosity is essentially the ability of a molecule or solid particle in a liquid to resist external stress or shear forces. The greater the viscosity of a solution, the greater its ability to withstand external stresses or shear forces [49]. The mixed liquor contains a large amount of viscous substances such as EPS, which makes it easy for the sludge flocs to adhere to the surface of the membrane, thereby accelerating membrane fouling, and reducing the gas-liquid flow rate generated by the aeration as well as forming a shearing effect on the membrane surface. This slows down the erosion of the contaminants on the membrane surface and worsens the operation of the membrane bioreactor [91].

Excessive viscosity of the sludge mixture increases the likelihood of sludge adhering to the membrane surface, thereby accelerating membrane fouling. In addition, Hu [92] showed that the sludge with high viscosity is not easy to clean after being adsorbed to the surface of the membrane, resulting in poor recovery of membrane flux.

#### 3.3.4. Effect of EPS and SMP on Membrane Fouling

**Figure 7:**
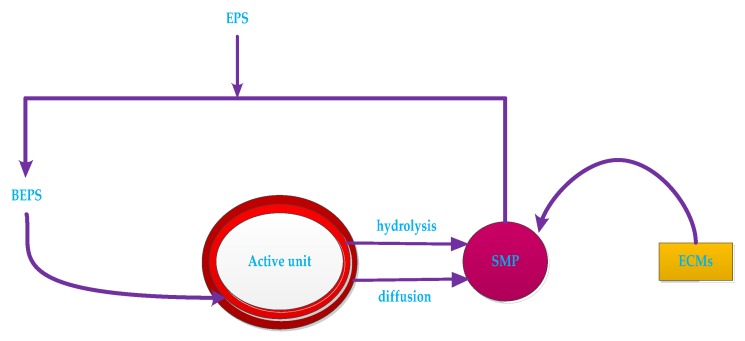
The relationship between EPS (extracellular polymers), BEPS (fixed EPS), SMP (soluble microbial product) active units and ECMs.

The effects of EPS and SMP on membrane fouling have received increasing attention in recent years. Figure 7 is a representation of the relationship between EPS, blend EPS (BEPS), SMP, active units (bacterial micelles and biofilm) and ECMs.

EPS includes insoluble organic matter secreted by cells, which is shed from the cell surface or caused by cell death. The main components are protein (EPSp) and carbohydrate (EPSc) [93]. The EPS in the sludge mixture and sludge floc is usually extracted by heating, organic solvent extraction and ion exchange. The EPS is also dissolved in the mixed liquor. It is a soluble microbial metabolite termed as soluble microbial product (SMP). EPS and SMP levels are typically characterized by CODcr, TOC, UV254 or directly by protein, polysaccharide and humic acid content [94].

EPS is an important factor affecting sludge settling performance and membrane fouling rate in MBR. Excessive EPS will further deteriorate the mutual flocculation effect between microorganisms and weaken microbial flocs [95]. The difference in EPS concentration of different activated sludge reflects the difference in the filtration performance of the corresponding sludge. The capillary sunction time (CST) value of the normal sludge is significantly smaller than that of the sludge bulking, which means that the filtration performance is good when the EPS concentration is low [96]. On the other hand, due to the high P/C ratio and hydrophobicity, the expanded sludge containing EPS is easily adhered to the surface of the membrane, thereby causing membrane resistance. At the same time, as the adsorption time prolonged, the membrane flux decreased significantly, indicating that the adsorption of EPS on the membrane surface will lead to irreversible fouling of the membrane [97]. Furthermore, EPS is a key factor affecting sludge agglomeration. The decrease of EPS causes the aggregation of sludge flocs to decrease, so that the size of sludge flocs becomes smaller, and smaller size flocs are easily deposited on the membrane surface. This will cause the fouling of membrane. Therefore, there should be an optimized EPS concentration in the membrane bioreactor, at which the floc structure of the sludge can be maintained, and the membrane fouling potential of the sludge flocs is minimized [98].

SMP is a large class of soluble organic matter produced by microbial metabolism, including polysaccharides, proteins, humic acids and nucleic acids. The composition is extremely complex and poor in biodegradability [99]. Due to the entrapment of the membrane, the adsorbed SMP accumulates on the surface of the membrane to cause concentration polarization, thereby causing membrane fouling. The formation of sedimentary layers is a major factor in membrane fouling [100]. During the formation of the sedimentary layer, SMP continuously fills the gap of the microbial flocs, making the deposited layer more dense, resulting in a decrease in the porosity of the deposited layer, resulting in a decrease in permeability and an increase in the specific resistance of the deposited layer. The higher the concentration of SMP in the sludge mixture, the denser the structure of the sediment layer and the smaller the void fraction. As the sedimentary layer continues to develop, the membrane flux decreases, which increases membrane fouling [101].

#### 3.3.5. The Effect of Microorganisms on Membrane Fouling

The biological phase in the MBR will change the sludge morphology, particle size distribution (PSD), EPS, viscosity and other parameters affecting membrane fouling. Compared with the traditional activated sludge method, the evolution of microbial populations in the MBR system is characterized by few species, which are vast and with obvious dominant populations. With the extension of the running time, the dominant populations in the MBR system consist of swimming ciliates, worms and bell worms, beetles, and red spotted worms. Membrane fouling can be predicted by dominant populations that indicate the state of the sludge [102].

The difference in biophase has a large effect on membrane filtration resistance. When the biophase is changed from a lower protozoan to a higher protozoa, it is reflected as a decrease in the starting point of the filtration resistance and slowing point in the growth rate of the microbial community. Wang et al. [103] showed that the micro-animals and activated sludge in MBR are a dynamic process of interaction. Meng et al. [104] found that filamentous bacteria play an extremely important role in membrane fouling during the operation of MBR. Excessive or too few filamentous bacteria can cause serious membrane fouling. The sludge flocs lacking filamentous bacteria are relatively fine, and it is easy to cause serious membrane pore blockage, and the activated sludge containing excessive filamentous bacteria will form a thick and firm filter cake layer, which increases the filtration resistance [105].

## 4. Membrane Fouling Control

### 4.1. Modification of Membrane Material Body

#### 4.1.1. Physical Blending

The physical blending is to physically mix the membrane material matrix with the modifying additive in a certain ratio, and the modified additive does not react with the bulk of the membrane material [106]. Physical blending modifications can balance the advantages and characteristics of the bulk membrane material and additives and have the advantages of both obtaining better cast film materials [107]. At present, hydrophilic materials blended with PVDF membrane materials can be roughly classified into two types. One is a hydrophilic polymer material, and the other is a small molecule inorganic particle. However, physical blending of membrane materials also has difficulties such as poor compatibility [108].

The advantage of blending modified hydrophilic polymer with PVDF polymer is that after adding relevant hydrophilic polymer to the film material, it can compensate for various performance defects of the film with raw material alone, and also can give the membrane material itself the new superiority [109]. In the polymer blend membrane, the compatibility between the polymers has a direct influence on the formation and structure during the phase separation process of membrane synthesis [110].

At present, in the improvement of the performance of the separation membrane, the polymers reported for blending with PVDF including polyvinylpyrrolidone (PVP), chloromethylated polysulfone (CMPS), polymethyl methacrylate (PMMA), poly vinyl acetate (PVAc), PEG, polyvinyl alcohol (PVA), sulfonated polystyrene (SPS), nylon 6, sulfonated polysulfone, polyacrylonitrile (PAN), polysulfone, and sulfonated polyaryl ether sulfone (SPES-C) [111].

Compared with hydrophilic polymer blending, blending small-molecular inorganic particles with PVDF to improve the hydrophilicity of the membrane is a rapid development in the recent years [112]. Commonly used inorganic particles are A1_2_O_3_, SiO_2_, TiO_2_, etc. The modified membrane prepared by using the blend casting solution perfectly combines the properties of the PVDF membrane with the hydrophilicity and heat resistance of the inorganic material to form a novel organic–inorganic composite membrane [113].

#### 4.1.2. Chemical Copolymerization

The chemical copolymerization modification can increase the hydrophilicity of the organic polymer film. This is carried out by adding a modified monomer to the original cast film material of the organic polymer film to cause a complicated copolymerization reaction and to form a new copolymer as a cast film material. Common methods for modifying membrane materials include copolymerization, block, chain extension, grafting, and so on. Sun et al. [114] induced the copolymerization of sulfonamide amphiphilic groups with acrylonitrile materials and modified the propylene amine groups to inhibit the protein adsorption properties and modified the polypropylene-sulfonamide copolymer membrane materials with excellent properties. The PVDF was modified with 10% NaOH to improve the hydrophilicity of the ultrafiltration membrane, and the hydrophilicity of the composite membrane obtained by grafting polyoxyethylene methacrylate (POEM) with 5 wt % to PVDF was also greatly improved [115].

Selina et al. [116] studied the factors that affect the performance and treatment efficiency of direct membrane filtration, and pointed out that membrane fouling is the main challenge of direct membrane filtration. Direct membrane filtration has been used as a promising technology for wastewater recovery and resource recovery in various laboratories and pilot scale studies, which is attributed to the advantages of direct membrane filtration process [117]. For example: (i) Direct membrane filtration processes have a relatively simple system configuration, requiring less capital cost and footprint. (ii) It has been well documented that direct membrane filtration of wastewater treatment could produce superior permeate quality that meets water discharge or reuse standards and effective concentration of nutrients for further recovery [118]. (iii) In some direct membrane filtration processes, water reclamation and resource recovery from wastewater can be simultaneously achieved, showing great potential for saving energy consumption, improving carbon neutrality, and minimizing footprint compared to conventional wastewater treatment processes [119].

### 4.2. Hydrophilic Modification of the Surface of Membrane Material

#### 4.2.1. Surface Coating

The surface coating modification method refers to coating a hydrophilic substance on the surface of the hydrophobic film to be modified, thereby improving the anti-fouling performance of the film. A comb polymer was coated on a PSF ultrafiltration membrane and the modified membrane was characterized by infrared spectroscopy and X-ray photoelectron spectroscopy (XPS). The presence of the coating layer was confirmed by the above characterizations [120]. Furthermore, it was found through experiments that the flux recovery rate of the modified membrane was significantly improved. In addition, when hydrophilic materials were coated on the surface of PVDF ultrafiltration membrane, it was found that the flux recovery rate of the modified membrane was more than 90% on the basis of improving the anti-fouling performance. Although the modification method is relatively simple in operation, the modified molecules coated on the film are easily detached from the surface of the film, and therefore long-term stable modification was unable to be achieved. Zhao et al. [121] first immersed the plasma-pretreated polyvinylidene fluoride (PVDF) membrane material in a 0.4% solution of TMC-hexane to form a modified PVDF-TMC membrane, and then immersed the modified membrane in sequence. The self-assembled coating modified layer was formed on the surface of the film with 0.5 wt % of SiO_2_-NH_2_ nanoparticle suspension and 0.1 wt % of SA solution. The experimental results showed that this method can effectively improve the anti-fouling performance [122].

#### 4.2.2. Membrane Modification by Low Temperature Plasma Surface Treatment

The plasma modification methods such as only plasma modify treatment (OPMT), plasma polymerization graft coating treatment (PPG-CT) and plasma trigger radical graft polymerization (PTRGP) on the surface of polymer materials can modify the membrane, but the modification mechanisms are slightly different. PTRGP method is the grafting of monomer on the surface of the membrane [123]. The copolymerization is mainly characterized by the fact that the monomer can penetrate into the pores of the membrane to carry out the grafting reaction, but the level of grafting is small. PPG-CT is mainly composed of the polymerization of monomer radicals, and its function is to provide high grafting rate. The morphology of the membrane surface changes greatly. The presence of polymer is obvious from the scanning electron micrograph of the membrane surface. OPMT is mainly caused by plasma ion incidence, plasma etching and plasma surface crosslinking. At high temperatures, the loss of membrane quality was more serious [124].

The low-temperature plasma treatment modification technology uses a gaseous substance such as an atom, a molecule or an ion in a state in which the positive and negative charges are in a plasma state to attack the polymer, and induces a chemical reaction such as hydrogen elimination on the surface of the membrane to introduce a large number of polar groups such as -OH, -COOH, -SO_3_H, -CO, and -NH_2_ [125]. This causes grafting of other monomeric substances on the surface of the membrane material to change its properties. The low-temperature plasma treatment modification technology can be realized in a medium to low temperature environment without changing the excellent characteristics of the original membrane. However, the instruments and equipment required for plasma treatment are generally costly and the operating conditions are harsh, thus limiting its use [126].

#### 4.2.3. Surface Grafting

Surface grafting refers to the modification of the thin layer on the surface of the membrane without changing the bulk properties of the membrane material so that a relatively stable chemical bond is formed between the membrane surface and the grafted polymer chain [127]. The effect of modification is more durable. Zhan et al. [128] prepared a CMPSF microporous membrane by phase inversion method, and introduced a large amount of primary amine groups on the surface of the membrane by chemical modification, thus constructing a surface initiation system -NH_2_/S_2_O_8_^2−^ The monomeric DMAEMA was successfully graft polymerized on the surface of the polysulfone microfiltration membrane to form a porous graft membrane PSF-g-PDMAEMA. Such a membrane can remove CrO_4_^2−^ ions in the water effectively by selective adsorption.

In recent years, research on membrane fouling control methods related to irradiation grafting has attracted more and more attention [129,130,131,132,133]. The reaction mechanism of UV irradiation grafting belongs to free radical reaction, which has low polymerization temperature and mild polymerization conditions. Some researchers have used ultraviolet radiation grafting to introduce acrylic acid into the polypropylene film, which greatly improved the hydrophilicity of the polypropylene film [129]. In the same way, the thio betaine methyl acrylate monomer was grafted onto the polypropylene microporous membrane, and the contact angle of the membrane was significantly reduced, and therefore the flux increased significantly [130]. In addition, the continuous hydrolysis of ultraviolet radiation was used to graft the pentaerythritol monoester to the surface of the polypropylene hollow fiber membrane, and the hydrophilicity and anti-fouling of the membrane were improved significantly [131]. It is also possible to graft hydroxyethyl methacrylate onto the surface of the polypropylene microporous membrane by ultraviolet light irradiation by using benzophenone and ferric chloride as co-initiators, so that the contact angle of the modified membrane is reduced [132].

Jin et al. [133] used polypropylene hollow fiber membrane as the matrix and sodium styrene sulfonate as the hydrophilic monomer to prepare a modified hollow fiber membrane with a different grafting rate under ultraviolet irradiation and infrared spectrum. The electron microscopy test proved that the modification was successful. When the graft ratio was 13.3%, the water contact angle of the film was 46°, which was significantly lower than that of the original film (64°) [134]. Up to a certain value, as the grafting rate was increased, the water flux increased. However, as the grafting rate increased further, the water flux decreased. When the grafting ratio was 9.0%, the water flux reached a maximum of 102 L/(m^2^·h), an increase of 29 L/(m^2^·h) compared to the original film. The hydrophilicity of the polypropylene hollow fiber membrane modified by ultraviolet irradiation improved significantly.

### 4.3. Optimization of Membrane Modules

**Figure 8:**
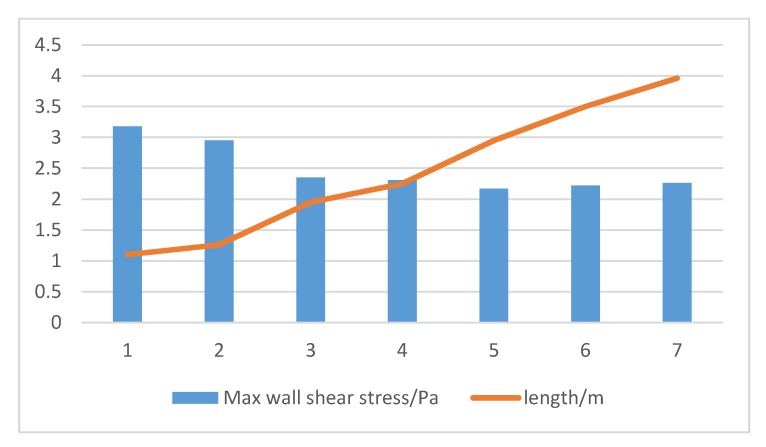
Effect of membrane module length on wall shear stress.

Factors that should be considered in the optimization of the membrane module are the shape of the membrane module, the placement of the module, hydraulic conditions, the diameter and the length as well as the tightness of the hollow fiber filaments [135]. As can be seen from Figure 8, the wall shear stress inside the membrane component decreased with the increase in the length of the filament.

Xiong et al. [136] used the Euler model and the porous medium model to calculate the fluid flow in the membrane module with different structures. The calculation shows that reducing the diameter of the aeration hole and increasing the number of aeration holes can promote the uniform distribution of gas–liquid two-phase flow field and liquid phase velocity field, as well as wall shear stress and turbulent viscosity enhancement [137]. Increasing the height of the membrane module is beneficial to increase the membrane area of a single membrane module while making full use of the gas for scrubbing during aeration. The liquid two-phase flow performs high-efficiency air scrubbing on the wall surface of the membrane. Xu [138] designed a novel spiral membrane module with a certain rotation angle by bionics principle, thereby improving the sensitivity of the membrane module to water, gas and other fluid disturbances, resulting in vibration of the membrane and increasing the elastic collision between the bubble and the membrane surface [139]. The concentration polarization phenomenon in the falling film separation effectively controls the membrane fouling, improves the membrane separation efficiency and flux, and reduces the energy consumption [140]. A Box–Behnken method can be used to optimize hydraulics of the membrane module. Multi-parameters such as inlet diameter, inlet length, membrane shell height, inlet/outlet end tube length, billet structure diameter, and inlet and outlet tilt angle can be used as variables in the experimental design. The Box–Behnken method is the synthesis of statistical design experiment technology. It uses the design of the Box–Behnken experiments and obtains data through the experiments to find the proper multiple quadratic equation which can fit the functional relationship between the factors and the effect value well. The optimal process parameters can be determined through the analysis of the regression analysis. The Box–Behnken method solves the multi-variable problem as a statistical method [141]. A membrane module design with optimal response variables can obtained by a series of experiments with varying membrane configurations. The particle residence time distribution and hydrodynamic characteristics of the liquid–solid two-phase flow in the three-dimensional model can be simulated by coupling the calculation between the Reynolds stress RSM turbulence model and the discrete phase model (DPM) based on the Euler–Lagrange algorithm [142]. The simulation results show that the velocity distribution of the shell surface of the cyclone-enhanced membrane module is more uniform and the shear stress of the membrane surface is high; the turbulent dissipation rate and vorticity distribution are different from those of the traditional membrane module [143]. The experimental results confirmed that the optimized membrane module has the characteristics of high yield of flux, low pressure drop and low membrane fouling rate [144]. To overcome the shortcomings of flat sheet membranes, a new type of folding membrane module has been designed. The vertical inclination and membrane spacing of the folding membrane module were optimized by constant pressure membrane filtration experiments, which greatly increased the maximum steady state membrane flux and the rate of transmembrane pressure rise. The reduction of the rate of transmembrane pressure rise is normal, and the removal rate of chemical oxygen demand (COD) and NH_3_-N is also improved [145]. Viet et al. found that, compared with the traditional membrane bioreactor, the osmotic membrane bioreactor (OMBR) has a broader application prospect in reducing membrane fouling and improving effluent quality [146]. Blandin et al. thought that the OMBR process is expected to consume less energy than MBR process, but further research is needed to confirm this [147].

### 4.4. Changing the Properties of the Feed Water

There are some factors affecting membrane fouling that exist in the feed liquid. In general, the direct method of controlling the characteristics of the feed liquid is to add a flocculant or adsorbent to the feed liquid. Compared with the monomer salt, the polymeric salt can provide more positive charge and electrically neutralize the suspended particles in the liquid, which can improve the removal rate of the suspended particles and increase the diameter of the particles [148]. The indirect method is to control the reaction by changing the sedimentation and flocculation performance of the feed liquid by adjusting the operating conditions such as HRT, SRT and reaction temperature.

Adding a commonly used adsorbent such as powdered activated carbon (PAC) to MBR can effectively control the development of membrane fouling, slow the rate of increase of TMP, and prolong the membrane operating cycle [149]. PAC has high adsorption capacity and can absorb dissolved organic matter, EPS, microparticles, etc. in the mixture, and can also be embedded in the sludge as a skeleton to form more solid sludge particles, which are not easily damaged by shear force and therefore will not release pollutants back into the bulk liquid. However, as the system operates, the PAC will gradually saturate, requiring regular replacement; therefore an optimum value for the dosage of PAC exists. Zeolite can also be an effective adsorbent. Zeolite can adsorb a part of sludge particles and reduce the resistance to membrane filtration [150]. However, zeolite also has an optimum dosage. Excessive zeolite will be adsorbed onto the surface of the membrane and increase the filtration resistance. The tiny particles brought along by the zeolite itself will increase the degree of membrane clogging and increase membrane fouling.

### 4.5. Control of Operating Conditions

The main operating conditions of MBR are membrane flux, operating pressure, aeration, pumping time and cleaning cycle. One measure commonly used in MBR is to control the membrane flux below membrane critical flux or sustainable flux operation. Zhang et al. [151] studied the short-term and long-term actual operation of the small-scale immersed MBR (SMBR) under constant flux and constant pressure modes of operation and found the constant pressure operation in the subcritical region and the constant pressure below the economic operating pressure TMP in the subcritical region was conducive to the long-term stable operation of the MBR. This is beneficial to the long-term stable operation of the MBR [152]. Membrane flux and membrane resistance have a great relationship with the shear force of membrane caused by the gas flow. In a certain range, the membrane flux will increase with the increase of aeration. When the aeration amount reaches a threshold, the flux will remain the same, and with further increase in the aeration flow rate, the membrane flux will decrease. This is because an excessive amount of aeration will break up the already flocculated suspended particles, making the particle size of the suspended particles smaller and more likely to block the pores of the membrane. Intermittent suction is also an effective measure to control membrane fouling. Zhang et al. [153] considered that the cleaning cycle and use time of the membrane had little effect on membrane fouling. In addition, ultrasonic irradiation can play a role in slowing down membrane fouling. Studies have shown that ultrasound has the effect of mitigating membrane fouling and can extend the membrane cleaning cycle [154]. The addition of ozone to the feed liquid can also effectively reduce membrane fouling. Wu et al. [155] showed that the optimum dosage (O_3_/SS) is 0.25 mg/g per day, which can reduce the EPS in the supernatant and enhance the suspended solids. In general, the control measures for membrane fouling are based on the factors that control membrane fouling. The common methods and principles of controlling membrane fouling are shown in Table 3. It is worth noting that the effect of a single control method is not ideal. In the actual application process, various methods should be combined according to the specific conditions to achieve the desired effect [156].

### 4.6. Cleaning of Membrane Fouling

Membrane cleaning can effectively remove and control membrane fouling, reduce TMP, and restore membrane flux [71]. The cleaning methods of contaminants in the MBR process can be divided into physical cleaning, chemical cleaning, and electric cleaning. In the actual operation process, it is difficult to achieve the best results with a single cleaning method. Generally, a combination of several methods will be effective in cleaning the membrane [157].

#### 4.6.1. Physical Cleaning

Physical cleaning mainly removes reversible contaminants in the membrane surface or membrane pores and the methods mainly include aeration, backwashing (air or filtrate), ultrasonication, sponge scrubbing and water washing. Physical cleaning allows the MBR to operate at a relatively constant flux without causing secondary contamination, but requires frequent cleaning at increased operating costs [47]. Aeration is the most commonly used membrane cleaning method in aerobic SMBR. It uses the cross-flow caused by the ascending airflow to reduce the deposition of particles on the membrane surface and flushes the membrane surface pollutants to reduce membrane fouling. In the operation of the hollow fiber membrane process, the role of aeration is to provide the oxygen demand for degrading organic matter, supply the oxygen demand for the growth and metabolism of the activated sludge itself, and remove contaminated deposits on the membrane surface [25]. A large amount of aeration produces a severe turbulent state on the membrane surface, and a strong shear force can carry away the filter cake layer deposited on the membrane surface. Intermittent operation and aeration combined can enhance the dispersion of pollutants attached onto the membrane surface and effectively retard membrane fouling. The suction is stopped after the membrane fouling occurs, and the continuous aeration of the membrane cake layer from the membrane surface can restore the flat sheet membrane flux [158]. However, there is also an optimal threshold for aeration.

Backwashing can remove most of the reversible pollutants and improve the membrane filtration performance. The key parameters affecting the backwashing effect are the backwash intensity, frequency, time and frequency-time ratio. Jiang et al. [159] found that low frequency, long time backwashing (600 s filtration/45 s backwashing) is more effective than high frequency, short time backwashing (200 s filtration/15 s backwashing). Fan et al. [160] obtained experimental methods and theoretical derivation for determining the optimal backwashing cycle of MBR, which can be used for automatic control of MBR backwashing. In the actual operation of MBR, cleaning by aeration and backwash are usually used in combination, which can achieve better results than a single method of cleaning.

Ultrasonic online cleaning methods can effectively control membrane fouling [13]. Ultrasonic waves have very special properties. The “cavitation” caused by the instantaneous release of ultrasonic energy concentrates on the solid–liquid interface, thus exerting a strong impact on the point of action and its surroundings and on the gel layer attached to the surface of the membrane. The precipitate produces a strong peeling effect [161]. However, excessive ultrasonic strength and time of action will break up the sludge flocs, affecting sludge activity and damaging the membrane module [99]. Therefore, the selection of appropriate ultrasonic intensity and time of action is essential for effective control of membrane fouling.

#### 4.6.2. Chemical Cleaning

Chemical cleaning is required when physical cleaning does not meet membrane fouling requirements. Commonly used chemical agents include alkali cleaning agents, acid cleaning agents, oxidizing cleaning agents, and surfactants (ethylenediaminetetraacetic acid EDTA, ammonium hydrogen fluoride, etc.) [162]. Alkali cleaning agents can effectively remove organic matter and biological foulants [163]. The process is as follows: inject water into the cleaning water tank, heat it with steam, start the cleaning pump, slowly add the cleaning agent, mix to make the cleaning agent completely dissolved, first clean the first section, then clean the second section for dynamic circulation for 40 min, and then soak for 50 min to clean alternately. When the pH is reduced by 0.5, add NaOH to control the pH value at 10–11. When the pH value is no longer reduced, carry out water washing. When the pH value of the effluent reaches 6–7, the water washing is finished [164]. An acid cleaning agent can effectively remove mineral and inorganic fouling [165]. During acid cleaning, water is injected into the cleaning water tank, heated by steam, the cleaning pump is started, hydrochloric acid is added slowly, the pH value is controlled at 2–3, and the cleaning is carried out in sections. The first section is cleaned, and then the second section is cleaned. The dynamic circulation is 40 min, and then the immersion is 40 min. In this way, the cleaning is carried out alternately. When the pH value is no longer increased, the water is washed [166]. When the pH value of the water reaches 6–7, the water washing is finished. Hydrochloric acid can remove hydrophobic organics better, while sodium hydroxide can remove more organic pollutants. The combination of the two can effectively remove the pollutants on the membrane surface, but the removal effect on the pollutants inside the membrane pore is poor [167]. Oxidizing cleaning agents can increase the hydrophilicity of organic polymer contaminants and can effectively remove the adhesion in the pores of the membrane. A surfactant can improve the contact of the cleaning agent with the pollutants, improve the cleaning effect, and it can also destroy the bacterial cell wall and weaken the foulants caused by the biofilm. Chemical cleaning can be used for both on-line cleaning and off-line cleaning, which can greatly restore membrane flux, but the cleaning waste can sometimes cause secondary fouling [168]. The four major factors to consider in chemical cleaning are: concentration of the cleaning agent, cleaning temperature, contact time, and mechanical strength of the film [169].

#### 4.6.3. Electric Cleaning

Electric cleaning achieves the effect of removing contaminants by applying an applied electric field on the film at a certain time interval to cause the contaminating particles to move away from the film surface in the direction of the electric field. However, this method requires that the film has a conductive function, or that the electrode can be mounted on the film surface, so that it is used less [170].

#### 4.6.4. Ultrasonic Cleaning

Ultrasonic irradiation can clean the fouled membrane through the production of important physical phenomena including microjet, microstream and shock waves [171]. Indeed, the particles can be released from the fouled membrane by the aforementioned physical phenomena taking place in a heterogeneous liquid–solid interface. Furthermore, the active hydroxyl radicals generated in the presence of ultrasonic irradiation can attack the adsorbed foulants and degrade the molecules of foulants which consequently result in membrane fouling control [172]. However, the membrane can be damaged through chemical reactions between the generated hydroxyl radicals and the membrane [173]. Therefore, the operational conditions should be optimized in ultrasound-MBR hybrid systems. The ultrasonic cleaning can be performed either in situ (online) or ex situ (offline) for cleaning the membrane of MBRs. Moreover, pretreatment of the wastewater by ultrasonic irradiation or by hybrid ultrasound methods prior to MBRs can decrease the organic loading of the wastewater and subsequently postpone the fouling of the membrane. Moreover, the ultrasonic method can be combined with other cleaning methods, i.e., chemical cleaning and backwashing, to improve the cleaning efficiency [174].

## 5. Conclusions

MBR technology is a highly competitive technology and has been widely used in various fields of wastewater reclamation and wastewater recycling. However, membrane fouling is a hindrance to the widespread promotion of this technology. Therefore, research on the causes, mechanisms and control technologies of membrane fouling is of vital importance to this technology. Although some achievements have been made, there is room for more improvements. The author believes that future research should focus on the following aspects:

(1) Due to the heterogeneous structure and complexity of WOM or the dissimilar fouling behaviors between different organic surrogates, it is highly recommended that use should be made of real wastewater in combination with advanced DOM analysis such as FT-ICR-MS (Fourier Transform-Ion Cyclotron Resonance-Mass Spectrometry), SEC-OCD, and CLSM, as well as online monitoring methods such as quartz crystal microbalance, or any visualization apparatus for exploring real-time organic membrane fouling formation. Real-time monitoring capabilities are also of great benefit for the optimization of the periodical cleaning of membranes during long-term FO processes. New data on the complex interactions between organic foulants and membrane materials can help the development of effective fouling control strategies for real wastewater. For instance, appropriate pretreatment can be developed/designed to effectively eliminate high molecular weight biopolymers (e.g., polysaccharides and proteins) to mitigate FO membrane fouling. Finally, considering the typical short-term formation of irreversible fouling in FO processes, the limited operational test period, and bench-scale nature of many previous studies, pilot-scale FO systems should be operated and studied with due provision for long-term monitoring if transition to successful full-scale wastewater FO processes is to be realized.

(2) An alternative solution for improvement of direct membrane filtration performance is to develop new membranes with increased anti-fouling properties. Several reported studies focused on developing the novel anti-fouling membranes and the lab-scale testing findings displayed their good performances in membrane fouling alleviation. In view of the absence of large-scale direct membrane filtration processes in the market, further research needs to be emphasized on (i) membrane fouling control technologies of direct membrane filtration, especially towards low energy consumption, less chemical usage, and easier operation and maintenance; (ii) development of novel membranes, especially having a mechanically robust nature with low-cost environmentally friendly materials and self-cleaning properties; (iii) comprehensive economic analysis, life cycle assessment, and carbon footprint analysis of different direct membrane filtration processes in order to identify the most suitable system configuration for further scale-up.

(3) The effect of the presence of ultrasound-active inorganic nanoparticles in the matrix of the membrane used in MBR systems can be investigated in future research. Ultrasound-active nanoparticles, i.e., ZnO, TiO_2_, etc., can produce hydroxyl radicals in the presence of ultrasonic irradiation. Generated hydroxyl radicals can degrade the foulants adsorbed on the surface of the membrane or captured in pores of the membrane, which consequently results in controlling the membrane fouling. High energy consumption of the ultrasonic transducers limits the application of ultrasound-MBR systems on a full scale. Further research is needed on the hybrid methods with low energy consumption for improving the application of ultrasonic technology in full-scale MBR systems.

(4) Aeration optimization, such as intermittent or cyclic aeration, automatic aeration control based on DO- or nutrient removal feedback and mechanically-assisted aeration scouring, has attracted much attention to achieve efficient membrane fouling control with less energy consumption. The method of aeration could be further optimized according to the CFD modeling on the fluidization and the scouring behavior of the particles in MBRs. Moreover, the attachment tendency of biofilm colonizers on the medium and membranes should be assessed. Moreover, chemical cleaning efficiency is highly related to the interaction between chemicals and foulants. The chemical reagent has greater potential to decrease the aging of membranes and even lead to the inactivation of microorganisms in the bioreactors. These adverse effects caused by the chemical cleaning will be highlighted in the future.

## Figures and Tables

**Table 1:** Onset of various fouling of membrane in a membrane bioreactor (MBR).

Fouling Type	Rate of Fouling (Pa.min^−1^)	Onset of Fouling
Reversible fouling	10–100	10 min
Irreversible fouling (removed by maintenance chemical cleaning)	1–10	1–2 weeks
Irreversible fouling (removed by mandatory chemical cleaning)	0.1–1	6–12 months
Non-restorable fouling	0.01–0.1	A few years

**Table 2:** Factors affecting membrane fouling.

Factor	Influence	Type of Wastewater
Membrane structure properties	The formation of the cake layer can be observed in the organic fouling, and inorganic fouling did not easily cause membrane fouling.	-
	The protein in the EPS was more than the polysaccharide, and the viscosity of the liquid increased.	Hot white pulp wastewater
Material characteristics	Increased SMP, increased filtration resistance, and deterioration of membrane due to fouling.	Domestic wastewater
	Supernatant SMP had more protein than polysaccharides, the viscosity increased, and the cake layer was easy to form.	Industrial waste
	When SRT increased, SMP and sludge viscosity increased.	Low concentration wastewater
Operating condition	At 30 and 50 d, the activated sludge floc increased, the low fouling rate SRT was too small, the SMP increased, and the fouling accelerated.	Municipal wastewater
	If it was too large, MLSS, SMP and other microbial products increased.	-
	HRT declined, protein substances in SMP increased, and EPS concentration increased.	Low concentration wastewater
	HRT decreased, filtration resistance increased, and granular sludge particle size decreased.	Artificial wastewater
	Small flocs increased under high temperature conditions, SMP, EPS increase, filter cake layer was easy to form	Evaporator condensate
	When the temperature went up, the membrane fouling resistance increased, and the protein content in EPS increased.	Hot pulping press

**Table 3:** Comparison of membrane fouling control methods.

Control Methods	Controlling Factors	Expected Results	Precautions
Modification of membrane material	Improve membrane surface hydrophilicity	Reduce the adsorption of impurities on the membrane surface and membrane pores	The membrane material should be modified according to treatment objectives
Optimization of membrane components	Improve membrane surface water conditions	Improve the effect of membrane surface gas flow flushing and decontamination	High mechanical properties for membrane materials
Aeration, ultrasound	Remove membrane deposits and improve liquid properties	Gas–liquid flow flushes out membrane deposits to increase activated sludge activity	Excessive aeration or microwave vibration will break up the sludge flocs and increase the fouling of the membrane
Add flocculant or adsorbent (PAC), ozone	Improve liquid properties	Improve sludge settling and reduce EPS and SMP in feed liquid	Inorganic flocculants change the pH of the feed, the adsorbent itself may also become a contaminant, and ozone inhibits microbial activity
Intermittent suction	Improve film surface detachment properties	Conducive to the membrane surface gas flow flushing with pollutants	Too long stoppage will affect the amount of water produced, too short to achieve the desired results

## Abbreviations

AnMBR	anaerobic membrane bioreactor
BEPS	blend extracellular polymer
BPC	biopolymer clusters
CFD	computational fluid dynamics
CFV	cross-flow velocity
CLSM	confocal laser scanning microscopy
CMPSF	chloromethylated polysulfone
COD	chemical oxygen demand
CST	capillary suction time
DO	dissolved oxygen
DMAEMA	dimethylaminoethyl methacrylate
DPM	discrete phase model
ECM	extracellular matrix
EDTA	ethylene diamine tetraacetic
EPS	extracellular polymers
F/M	food to microorganism ratio
FO	forward osmosis
HA	humicacid
HPI	hydrophilic
HRT	hydraulic retention time
HPO	hydrophobic
MBR	membrane bioreactor
MF	microfiltration
MLSS	mixed liquid suspended solids
OMBR	osmotic membrane bioreactor
OPMT	only plasma modify treatment;
OLR	organic loading rate
PAC	powdered activated carbon
PAN	polyacrylonitrile
pH	hydrogen ion concentration
PE	polyethylene
PES	polyethersulfone
POEM	polyoxyethylene methacrylate
PPG-CT	plasma polymerization graft coating treatment
PTRGP	plasma trigger radical graft polymerization
PVDF	polyvinylidene fluoride
PSD	particle size distribution
PVC	Polyvinyl chloride
PVP	Polyvinyl pyrrolidone
PS	polysulfone
PVAc	polyvinyl acetate
PVA	polyvinyl alcohol
SA	sodium alginate
SEC-OCD	size exclusion chromatography with organic carbon detector
SF-DMBR	self-forming dynamic membrane bioreactor
SMB	sponge-based moving bed
SMBR	small-scale immersed MBR
SMP	microbial metabolites products
SRT	sludge retention time
SG	suspended-growth
SPS	sulfonated polystyrene
SPES-C	sulfonated polyaryl ether sulfone
TMP	transmembrane pressure
TOC	total organic carbon
UV	under voltage
WOM	wastewater organic matter
XPS	X-ray photoelectron spectroscopy

## Appendix A

Table A1 shows the review papers on membrane fouling in specific areas in recent years.

**Table A1:** Review papers on membrane fouling in specific areas in recent years.

Research Area	References
FO; RO; Driven membrane processes; Biofilm dynamics; Membrane performance; Concentration polarization	[2,12,26,56,105]
EPS; SMP; Microbial community structure; Microbial flocs; Microbial soluble substances; Membrane modification	[7,8,15,34,63]
Membrane cleaning; Membrane fouling control; Cross-flow membrane filtration; osmotic pressure	[2,4,10,11,13]
Inherent properties of membrane; Operating conditions; Mixed liquid properties; Fouling mechanisms	[14,22,34,81,88]
Anaerobic membrane bioreactor; Influencing factors; Domestic wastewater; Biosolids production; Energy; Reuse	[23,30,43,45,47,166]
Chemical oxygen demand; SRT; HRT	[22,24,37,60]
Ultrasonication; Hollow fiber membrane; Mathematical model; Emerging micropollutants	[27,44,60,96]
Nutrient recovery; Phosphate recovery; Ammonia recovery; Hybrid system; Direct membrane;	[22,60,112,116,128]
